# Longitudinal Associations between Intake of Fruit and Vegetables and Height Attainment from Preschool to School Entry

**DOI:** 10.3390/ijerph18116106

**Published:** 2021-06-05

**Authors:** Rafaela Rosário, Mina Nicole Händel, Jeanett Friis Rohde, Nanna Julie Olsen, Berit Lilienthal Heitmann

**Affiliations:** 1 School of Nursing, University of Minho, 4710-057 Braga, Portugal; 2 Health Sciences Research Unit: Nursing (UICISA: E), Nursing School of Coimbra (ESEnfC), 3004-011 Coimbra, Portugal; 3 Research Unit for Dietary Studies, The Parker Institute, Bispebjerg and Frederiksberg Hospital, DK 2000 Frederiksberg, Denmark; mina.nicole.holmgaard.handel@regionh.dk (M.N.H.); jeanett.friis.rohde@regionh.dk (J.F.R.); nanna.julie.olsen@regionh.dk (N.J.O.); Berit.Lilienthal.Heitmann@regionh.dk (B.L.H.); 4 The Boden Institute of Obesity, Nutrition, Exercise & Eating Disorders, University of Sydney, Sydney, NSW 2050, Australia; 5 Department of Public Health, Section for General Practice, University of Copenhagen, DK 1014 Copenhagen, Denmark

**Keywords:** children, fruit and vegetable, height

## Abstract

To examine associations between fruit and vegetable intake in young childhood and height attainment during preschool and at school entry. Data for this study was based on “The Healthy Start” primary intervention study, which included 635 obesity-prone children, (58% boys), from the greater Copenhagen area, with a mean (SD) age of 4.0 (1.1) years (age range 2–6 years) at baseline. In the current study, 553 children (57% boys) were included with information on dietary intake at baseline and height measured at baseline (preschool age), and 511 children (56.8% boys) with the height measured at school entry (~6 years old). Height was measured by trained health professionals during the intervention and by school nurses at school entry. Information on intakes of fruit and vegetables, separately and combined, was gathered with four-day dietary records reported by parents. Participants were grouped into tertiles for their intakes at baseline. Compared to boys with low consumption, those with a moderate and high intakes of fruit and vegetables (F&V) had a greater attained height at preschool of 1.3 cm (95% confidence interval (CI): 0.3; 2.3) and at school entry of 2.4 cm (95% CI: 0.8; 3.9) and 1.8 cm (95% CI: 0.2; 3.4), respectively, also after adjustment for differences in age, body mass index (BMI), and total energy intake. Additional adjustment for mid-parental height and parents’ education did not alter the significant associations between moderate consumption of F&V and attained height at preschool and school entry. There was no association among girls. Our results showed that a moderate consumption of F&V was directly associated with higher attainment in height at preschool and school entry in boys. From a public health perspective, it should be prioritized to continue developing intervention programs to improve fruit and vegetable intake.

## 1. Introduction

A substantial number of epidemiologic studies show that early childhood growth influences the development of later health and disease (DOHaD), including the risk of later childhood or adult metabolic, cardiovascular, endocrine, and cognitive conditions [1,2,3]. Moreover, there is a growing interest in early life genetic, early exposures, environmental (e.g., nutrition), and social determinants of childhood growth [4]. These determinants may counter growth and development during childhood and adolescence or, when healthy, strengthen the gains from early childhood and correct inadequacies [4,5]. Anthropometric measures, such as height and body-mass index (BMI), are proxy variables of the quality of nutrition and the living environment during childhood and are associated with health outcomes during adult life [6,7,8].

When exclusively examining gain in height, family, and twin studies show that heritable factors are essential [9]. Nevertheless, the variations in height across populations and over time can only partly be explained by genetic factors [10,11,12,13,14]. We know that the first years of life are crucial for growth [15]. Adversities during these years (e.g., poor nutrition) might have irreversible consequences, leading to poor growth and development [4,15]. Nevertheless, evidence also suggests that accelerated linear growth during childhood and adolescence (catch-up growth) can arise [16] as a result of healthy nutrition and living conditions [4,17,18]. Indeed, nutrition, and not least under-nutrition, are important non-genetic determinants affecting growth and stunting [10,11]. A former longitudinal study found that children at 21 months who consumed a larger meal size had a faster growth rate [19]. Because energy is needed for growth and development, the growth in the body size increases energy needs. Some nutrients, such as protein [20,21], iron, zinc, and vitamin-A, have strong relationships with growth [11,22]. Additionally, certain foods are considered important for growth, including milk consumption that has been shown to influence height attainment in adulthood [23] and during preschool [24]. It is also well known that fruit and vegetable consumption affect satiety, food intak, and body weight [25,26]; however, less attention has been given to understand the importance of these foods for growth and height attainment. Still, evidence supports that early life eating behaviors are related to food preferences later in life [27,28]. 

The biologic rationale behind the importance of fruit and vegetable consumption for growth in height is not fully explained but may depend on the influence of F&V on bone mass and growth [29,30,31]. First, fruit and vegetables have important roles in the acid-base balance. High consumption of fruit and vegetables, which provide alkaline products, may exert a buffering effect in metabolic acid load (protein-rich foods, grains, and cereals) and hence may be of importance for skeletal health and growth [32]. Furthermore, the high potassium and magnesium content in fruit and vegetables can potentially contribute to the sparing of bone tissue and retainment of calcium [33]. Fruit and vegetables are rich in vitamins, such as vitamin C [34] and vitamin K [35], both of which have been found to have beneficial effects on bone health. Other micronutrients such as zinc have also been related to growth in height [36]. Current dietary recommendations of fruit and vegetables for children aged 3–6 years old are well established, although they vary between countries. For example, in the USA, 1 to 1.5 cups (~250 to 375 g) of fruit and vegetables is recommended [37], while in Australia the amount is 150 to 338 g [38], and in Denmark a minimum of 300 g of fruit and vegetables is recommended [39]. Nonetheless, we are not aware of any previous studies examining whether fruit and vegetable consumption may be related to height development during early childhood. Hence, we aim to fill this knowledge gap by investigating the associations between fruit and vegetable intake and height development during preschool (<6 years old) and at school entry (~6 years old). 

## 2. Materials and Methods

### 2.1. Participants

The present study is derived from “The Healthy Start” project, a Danish cohort of children followed prospectively. Although the full description of the project is reported elsewhere [40], briefly, it comprised children born between 2004 and 2007 in 11 selected municipalities from the greater Copenhagen area. To be eligible for the study, the child must be considered susceptible to obesity by one of the following criteria: high birth weight (>4000 g) or mothers with a high pre-pregnancy BMI (>28 kg/m^2^). Information on birth weight and maternal pre-pregnancy BMI was obtained from the Danish Medical Birth Registry. Furthermore, a subgroup from one municipality was selected based on low maternal education (<10 years of schooling). Information on the maternal educational level was obtained from administrative birth forms. A total of 5902 children met these criteria and were eligible to be enrolled in the study. Exclusion criteria included children who emigrated or moved away from the municipality where they were born, who had no permanent address, lived at a children’s home, or had requested protection from participation in statistical or scientific surveys based on data delivered from the Danish Central Person Registry. In total, 3058 children were eligible and randomized into either an intervention group, control group, or shadow control group. Parents of children in the intervention and control groups were sent an invitation to participate (*n* = 3058). Of these, 635 agreed to participate. No incentives were offered for participation. In the current study, we included those 553 children with full information on anthropometric and dietary information at baseline, of whom 511 children also had anthropometric measures taken at school entry. 

### 2.2. Anthropometrics

During the health examinations at baseline, each child’s height and weight were measured to the nearest 0.1 cm and 0.1 kg using a stature meter (Soehnle 5002 or Charter ch200P) and a mechanical weight or a beam scale (TanitaBWB-800 or SV-SECA 710), respectively. For both measurements, the child was barefoot and stood on the scale with light clothes only. BMI was computed as the ratio of weight (kg) and the squared height (m^2^). The measurements at baseline were taken by trained trial personnel. Standardized BMI (BMI SDS) was calculated using the LMS method [41]. 

The height at follow-up was gathered by school nurses as part of the nationwide routine health assessment in 1st grade, performed at the local schools. The anthropometric measurements are required to follow standardized criteria established by the Danish Health Authority. Thus, the child is measured without shoes on, standing with legs together against a wall and looking straight ahead (“Frankfort plane”). The measurement is made with a vertical trailing edge. The child must not lean on the wall or touch anything during the measurement [42]. Participating children at baseline were manually linked with the Central Personal Registry number to the school-health records (heights and weights) measured at enrolment (~6 years old). Additionally, age at school entry was gathered by school reports.

### 2.3. Fruit and Vegetables Intake

Parents filled out a four-day dietary intake record for their child from Wednesday to Saturday at baseline. Portion sizes were estimated based on a picture book that accompanied the record. Calculation of nutrients and total energy from the dietary record was performed by the software program Dankost 3000. Children were divided according to tertiles of total energy intake as follows: (i) low-bottom tertile (total energy intake ≤ 4.4 MJ/day); (ii) moderate-middle tertile (4.4–5.1 MJ/day), and (iii) high-top tertile ≥ 5.1 MJ/day). Fruit and vegetable consumption, in grams, was computed as the total amount of fruit, vegetables, and fruit and vegetables combined (F&V). Fruit included fresh, canned, and frozen (excluding jam, fruit juice, dried fruit, and fruit products with added sugar). Vegetables included fresh, canned, and frozen (excluding fried onion, ketchup, pickles, and potatoes). Children were divided according to the tertiles of the amount of consumption in each food group: (i) low-bottom tertile (fruit ≤ 61 g/day; vegetable ≤ 67 g/day; F&V ≤ 145 g/day), (ii) moderate-middle tertile (fruit 61–110 g/day; vegetable 67–113 g/day; F&V 145–216 g/day), and (iii) high–top tertile (fruit ≥ 110g/day; vegetable ≥ 113 g/day; F&V ≥ 216/day).

### 2.4. Other Measurements

Information about age, sex, and municipality of residence was retrieved from the Danish Medical Birth Registry. Socioeconomic status indicated by education, which has previously been associated with adult height [11], was obtained at baseline with a questionnaire completed by parents. Parents’ education was computed in the current study into the following categories: (i) maximum of 10th grade of education for at least one parent, (ii) minimum of 10th grade of education for one parent, and (iii) minimum of 10th grade of education for both parents. With the same questionnaire, self-reported parental height was gathered. Parental height was calculated as the average of father´s and mother´s heights. A new variable was computed (mid-parental height) as above and below the median of parental height. This has been suggested to be a better indicator of the genetic potential to influence on child´s height than a parent´s individual heights [43].

### 2.5. Ethical and Legal Requirements

The study was in accordance with the Helsinki II declaration and was approved by the Danish Protection Agency (present number: 2015-41-3937; previous number: 2007-41-0530). According to Section 2. (1) of the Danish Act on a Bioethics Committee System and the Processing of Bioethics Projects, the project was defined not to be a bioethics project and, as a result, did not need approval from the Scientific Ethical Committee for the Capital Region of Denmark (14 May 2007, Journal no.: H-A-2007-0019). All parents of the participating children filled in the informed written consent. In addition, the study was registered, retrospectively, in ClinicalTrials.gov, ID NCT01583335, on 31March 2012. 

### 2.6. Statistical Analysis

Descriptive statistics are presented as mean and standard deviations (SD) and percentages according to the type of variables. Differences between subjects at baseline were analyzed using t-test for continuous variables and chi-squared for percentages. 

Associations between fruit and/or vegetable consumption at baseline (during preschool) and attained height during preschool or at school entry (dependent variables) were performed using generalized linear models (GLM). The residuals are normally distributed. The models were further adjusted for age, BMI SDS, and total energy intake at baseline (Model 1), along with mid-parental height and parental education (Model 2). As this report is part of an intervention study, we had the following considerations. First, a previous study found that the Healthy Start intervention had no effect on F&V intake [44]. Second, we analyzed the effects of the intervention program on height development, and because no effects were found, the results are shown without an additional adjustment for allocation group (control or intervention). Third, we conducted the same analysis in the control group only, and found essentially similar results. Finally, because there were significant interactions between fruit and/or vegetable intake and height development for boys and girls, the final analyses were performed separately by gender. Data analyses were performed using SPSS, version 26.0 (SPSS Inc. Chicago, IL, USA), with a 0.05 level of significance considered.

## 3. Results

The characteristics of the participants at baseline are presented in Table 1. There were differences in total energy intake (*p* < 0.001), fruit consumption, and the anthropometric variables (*p* < 0.05) between girls and boys. Boys had significantly higher total energy intake, fruit consumption, weight, and height than girls. 

Regarding fruit intake, the first tertile had a mean intake (SD) of 37.6 (17.0) g of fruit, 40.1 (17.9) g of vegetables, and 101.8 (33.1) g of F&V. Children in the second tertile had a mean intake of 85.6 (13.5) g of fruit, 88.6 (13.4) g of vegetables, and 179.3 (20.9) g of F&V. Finally, children in the third tertile had intakes of 160.8 (45.7) g of fruit, 164.4 (52.4) g of vegetables, and 295.9 (63.7) g of F&V (data presented in a supplementary Table S1).

Children at baseline had a mean height of 104.3 (9.5) cm, a weight of 17.9 (3.2) kg, and a BMI of 16.3 (1.3) kg/m^2^. At follow-up, children had 123.5 (5.6) cm of height, 24.8 (3.5) kg of weight, and a BMI of 16.3 (1.4) kg/m^2^.

After adjusting for age, BMI SDS, and total energy intake (Model 1), in boys, both a moderate and high F&V consumption at baseline were each directly associated with a 1.3 cm (95% CI 0.3; 2.3) greater attained height during preschool. Additionally, in boys, both a moderate and a high F&V intake (when compared with the reference low consumption) were directly associated with 2.4 cm (95% CI 0.8; 3.9) and 1.8 cm (95% CI 0.2; 3.4) greater attained height at school entry.

Results from the model that also adjusted for mid-parental height and education (Model 2) indicated that, in boys, a moderate but not a high F&V intake at baseline was directly associated with 1.4 cm (95% CI 0.2; 2.7) greater attained height during preschool and 1.8 cm (95% CI 0.03; 3.6) at school entry (Table 2).

## 4. Discussion

In this prospective study, compared to boys with low intakes of F&V, those boys with moderate and high intakes attained greater height in both preschool and at school entry, even after adjustment for differences in age, BMI SDS, and total energy intake. Additional adjustment for mid-parental height and parents’ education did not alter the significant associations between moderate F&V intake and attained height at preschool and school entry. Nevertheless, the associations between high consumption of F&V and attained height during preschool and at school entry were no longer significant. To the best of our knowledge, this is the first study to examine associations between fruit and vegetable intake and attained height during preschool and at school. 

Earlier studies found associations between some nutrients, such as protein [20,21], vitamins A and D, and linear growth [11]. Furthermore, a high milk consumption was previously found to be directly associated with height in preschoolers [24] and adults [23]. In the current study, and after doing a sensitivity analysis having milk intake, energy from protein intake, and calcium intake as additional confounders, similar significant associations between F&V and height attainment were found. We are not aware of any previous studies that analyzed associations between intake of fruit and vegetables and development in height in early childhood, but some previous studies found inverse associations between fruit and vegetables and BMI [45]. This may potentially be explained by the low energy density, the low content of fat, and the high content of water in a diet high in fruit and vegetables, along with the high content of dietary fiber associated with high satiety [25]. Although BMI is an index of mass that accounts for height, it is possible that children with a high consumption of fruit and vegetables have an overall rich and more healthy nutritional pattern [46], both adding to axillary growth and maintaining leanness. On the other hand, our group previously found that greater adiposity among preschoolers was associated with higher attained height at school entry [47]. As we found direct associations between intake of fruit and vegetables and height attainment, also after adjusting for BMI SDS, the latter does not seem to be the mediator of the currently found relationship.

There were significant interactions between fruit and/or vegetable intake and height attainment for boys and girls. Some of these differences may start at birth and remain generally small until puberty when the girls start pubertal growth spurts before the boys [48]. However, from early infancy and through pre-puberty, sex-differences are also observed for fat mass [49] and hormone levels that are implicated in dietary behaviors and metabolic processes (e.g., insulin) [50]. Environmental and social determinants of health, such as the availability of unhealthy foods in the home or exposure to family conflicts, are associated with a low nutritional dietary practice, such as the consumption of sweet snacks or take-away, in girls more so than in boys [51]. Although we did not find differences in the reporting of overall intake of fruit and vegetables among boys and girls, we do not exclude possible gender-differences in reporting other foods, including low nutritional and energy-dense foods and beverages. Nevertheless, we adjusted the model for total energy intake in order to avoid the effect of calories in obtaining gains in height. Results from the current study also indicate that, when adjusting for mid-parental height and education, in boys, only a moderate, and not high, F&V intake at baseline is directly associated with greater attained height during preschool and at school entry. It is known that height has both genetic [9,43] and environmental influences [10]. However, to date, the reasons for the heterogeneity of human growth among children, even in the same age group, are unclear. Not surprisingly, in our study, children´s height is significantly associated with mid-parental height. Nonetheless, this explanation does not fully explain our results. The similarity in height between low and high consumers of F&V is not attributed to differences in age, BMI SDS, or total energy intake, as all were controlled for in the analysis. Hence, we do not exclude that children with a moderate consumption of F&V also have a higher variety and an overall better diet quality [46]. In addition, it is possible that there is not a dose-response effect of F&V intake on attained height, but an effect up until a certain (threshold) level of F&V intake. Finally, taller parents may perceive their children as “tall” or as “growing well”, and may not encourage them to consume as much healthy food, such as F&V, as they do their smaller counterparts. Further research should examine how parental perceptions of child growth is related to F&V intake and diet quality pattern. 

Our study has important strengths and limitations. Important strengths include the prospective study design, the fact that height development was measured by trained personnel and by standardized procedures, and that fruit and vegetable consumption was obtained with a four-day dietary intake record, establishing a typical dietary intake. Furthermore, we adjusted the analyses for potential confounders, such as age, BMI, total energy intake, mid-parental height, and parents’ education, which can account for part of the variation in attained height [10]. We know that height trajectories are highly variable across countries, which indicates heterogeneous health advantages [52]. Parental education, a socioeconomic determinant, has been associated with both stunting and dietary intake patterns, and was therefore considered relevant in the context of the dietary patterns and height relations [53]. Finally, we analyzed fruit and vegetables both combined and separately. We know that fruit and vegetable recommendations vary according to country [37,38,39]; nevertheless, these guidelines see all types of fruits or vegetables similarly, although they are heterogeneous in their nutritional properties [54,55]. 

We consider one limitation to the study to be the somewhat limited sample size, partly reflecting low respondent motivation and the burden of being involved in dietary studies. Future studies might use flexible strategies in recruiting participants (e.g., times available for the interview or a second call if a person does not show) in order to obtain a higher sample size. Additionally, although the focus on obesity susceptible children in the Healthy Start study was not expected to influence height for age attainment, this focus still limits the generalization of the results to non-susceptible children. Reporting bias may, as in other dietary studies, be a problem, even though diet intake was assessed using a validated tool. Generally, 98% of all Danish children between the ages of 1 and 6 attend early childhood education and care centers. Those children with 3 years old and over attend these centers on average for 32.7 h per week [56]. The parents recorded the four-day dietary record from Wednesday to Saturday, and information was not available on the fruit and vegetable consumption at kindergartens. Although total intake of fruit and vegetables was likely underestimated, F&V provided by parents may have been accurately reported, even if further underreporting can still not be excluded. Provided this underreporting was random, it would have attenuated our results. Attenuation would also result if those with recorded low intakes under reported most, and hence we may have overlooked potential associations.

Our results show that, compared to boys in the lowest tertile of F&V intake, those with moderate intakes attained more height growth. Although fruit and vegetable intake is close to Danish recommendations, our results do not demonstrate levels of adequate or inadequate intakes of F&V, but rather that mean intakes of 180.3 (20.0) g F&V were related to a higher average attained height. Finally, all the participating children were of Danish origin. Denmark is considered a generally highly affluent country, thus potentially limiting the generalization of our results to children from less affluent contexts. Furthermore, a previous study with this population showed that the children generally follow the Nordic nutrition recommendations and have an overall good diet quality index [44]. 

Height is a simple indicator of health and is easy to routinely collect, and there is growing evidence showing its association with current and future population health, nutritional status, and development [10]. Hence, to achieve the full potential of attained height, it may be relevant to secure adequate consumption of fruit and vegetables early in life. Furthermore, the quality of intake and their preparation are additional strengths for future studies.

## 5. Conclusions

A moderate and high F&V intake was associated with a greater height attainment in boys at preschool and at school entry, also after adjustment for differences in age, BMI SDS, and total energy intake. The relationship with attained height at both preschool and school entry persisted after further adjustment for mid-parental height and parents’ education for moderate, but not for high, consumption of F&V. From a public health perspective, a priority will be to develop intervention programs to secure adequate fruit and vegetable intake and to follow young children for their height development. Follow-up studies should explore further associations of dietary patterns, lifestyles, and steep weight trajectories and attained height during puberty and into adulthood.

## Figures and Tables

**Table 1:** Participants characteristics at baseline.

	All *n* = 553	Boys *n* = 314	Girls *n* = 239	*P*
Age (years)	4.0 (1.1)	4.0 (1.1)	3.9 (1.1)	0.4
Height (cm)	104.4 (9.2)	105.2 (9.0)	103.2 (9.5)	0.01
Weight (kg)	17.9 (3.2)	18.2 (3.0)	17.5 (3.5)	0.01
BMI-SDS	0.4 (0.9)	0.4 (0.9)	0.3 (0.9)	0.03
Total energy intake (MJ/day)	4.8 (1.0)	5.0 (1.0)	4.6 (1.0)	<0.001
Fruit (g)	94.8 (58.4)	99.9 (58.3)	88.1 (57.9)	0.02
Vegetables (g)	97.9 (60.7)	94.7 (59.7)	102.0 (61.8)	0.2
Fruit and vegetables (g)	192.7 (90.4)	194.6 (90.2)	190.1 (90.8)	0.6
Mid-parental height (m)	1.8 (0.05)	1.8 (0.05)	1.8 (0.05)	0.6
	*n* (%)	*n* (%)	*n* (%)	
Total energy intake				<0.001
Low	184 (33.3)	82 (14.8)	102 (18.4)	
Moderate	185 (33.5)	108 (19.5)	77 (13.9)	
High	184 (33.3)	124 (22.4)	60 (10.8)	
Education				
Maximum 10th grade at least one parent	8 (2.3)	5 (1.4)	3 (0.9)	0.1
Minimum 10th grade for one parent	33 (9.4)	11 (3.1)	22 (6.3)	
Minimum 10th grade for both parents	309 (88.3)	177 (50.6)	132 (37.7)	
Fruit intake				0.002
low	184 (33.3)	86 (15.6)	98 (17.7)	
moderate	185 (33.5)	119 (21.5)	66 (11.9)	
high	184 (33.3)	109 (19.7)	75 (13.6)	
Vegetables intake				0.5
low	184 (33.3)	110 (19.9)	74 (13.4)	
moderate	185 (33.5)	100 (18.1)	85 (15.4)	
high	184 (33.3)	104 (18.8)	80 (14.5)	
F&V intake				0.9
low	184 (33.3)	105 (19.0)	79 (14.3)	
moderate	185 (33.5)	103 (18.6)	82 (14.8)	
high	184 (33.3)	106 (19.2)	78 (14.1)	

All variables are expressed as mean (SD) unless stated otherwise. Results from t-test in continuous variables and Fisher’s exact test for nominal and ordinal variables. Not all individuals were measured for these variables. Results based on data mid-parental height (*n* = 306 and 235 for boys and girls, respectively) and parental education (*n* = 193 and 157 for boys and girls, respectively).

**Table 2:** Results of generalized linear models for the association between fruit and vegetables consumption and height (cm) at preschool and school entry.

				Fruit Intake			Vegetables Intake			F&VIntake	
			Low	Moderate	High	Low	Moderate	High	Low	Moderate	High
Attained height during preschool	Boys	Unadjusted *n* = 314	ref.	−1.1 (−3.6; 1.3)	−0.5 (−3.0; 1.3)	ref.	−0.2 (−2.7; 2.2)	1.2 (−1.2; 3.6)	ref.	−0.6 (−3.1; 1.8)	0.4 (−2.0; 2.8)
		Model 1 *n* = 314	ref.	−0.6 (−1.6; 0.3)	0.01 (−1.0; 1.0)	ref.	0.3 (−0.7; 1.2)	0.7 (−0.2; 1.7)	ref.	1.3 (0.3; 2.3) *	1.3 (0.3; 2.3) *
		Model 2 *n* = 188	ref.	−0.4 (−1.6; 0.8)	0.2 (−1.0; 1.5)	ref.	0.2 (−1.1; 1.4)	0.2 (−1.0; 1.4)	ref.	1.4 (0.2; 2.7) *	0.7 (−0.5; 1.9)
	Girls	Unadjusted *n* = 239	ref.	−0.5 (−3.2; 2.5)	0.4 (−2.5; 3.2)	ref.	1.4 (−1.4; 4.3)	4.5 (1.6; 7.5) *	ref.	1.5 (−1.4; 4.4)	2.9 (−0.1; 5.9)
		Model 1 *n* = 239	ref.	−0.4 (−1.6; 0.7)	0.2 (−0.9; 1.4)	ref.	0.2 (−1.0; 1.3)	0.3 (−1.0; 1.5)	ref.	−0.8 (−1.9; 0.4)	−0.04 (−1.2; 1.1)
		Model 2 *n* = 155	ref.	0.2 (−1.0; 1.5)	0.5 (−0.8; 1.7)	ref.	0.2 (−1.0; 1.4)	−0.1 (−1.4; 1.2)	ref.	−0.01 (−1.2; 1.2)	−0.1 (−1.4; 1.2)
Attained height at school entry	Boys	Unadjusted *n* = 290	ref.	−0.5 (−2.1; 1.1)	0.1 (−1.5; 1.1)	ref.	1.0 (−0.5; 2.6)	1.4 (−0.2; 2.9)	ref.	2.4 (0.9; 3.9) *	2.1 (0.6; 3.6) *
		Model 1 *n* = 290	ref	−0.7 (−2.2; 0.9)	−0.3 (−1.9; 1.4)	ref.	0.6 (−0.9; 2.2)	1.1 (−0.5; 2.7)	ref.	2.4 (0.8; 3.9) *	1.8 (0.2; 3.4) *
		Model 2 *n* = 171	ref.	0.03 (−1.8; 1.8)	0.3 (−1.6; 2.2)	ref.	0.1 (−1.8; 1.9)	0.2 (−1.6; 2.1)	ref.	1.8 (0.03; 3.6) *	0.7 (−1.2; 2.5)
	Girls	Unadjusted *n* = 221	ref.	−0.7 (−2.6; 1.2)	0.2 (−1.5; 1.9)	ref.	1.1 (−0.8; 3.0)	0.5 (−1.4 2.4)	ref.	−0.2 (−2.1; 1.7)	−0.4 (−1.5; 2.4)
		Model 1 *n* = 221	ref.	−0.7 (−2.4; 1.0)	0.1 (−1.5; 1.8)	ref.	1.1 (−0.7; 2.9)	0.1 (−1.8; 1.9)	ref.	−2.5 (−2.0; 1.5)	0.2 (−1.7; 2.1)
		Model 2 *n* = 147	ref.	0.2 (−1.8; 2.2)	1.0 (−1.0; 3.0)	ref.	1.4 (−0.6; 3.4)	0.5 (−1.7; 2.7)	ref.	0.7 (−1.2; 2.6)	0.3 (−1.8; 2.4)

Notes: Model 1—adjusted for age and BMI SDS at baseline and total energy intake. Model 2—Model 1 plus mid-parental height and parents’ education. All values are B (95%CI). * *p ≤* 0.05.

## Data Availability

In order to protect sensitive patient information, all data has been deposited in The Danish National Archives and is available upon online request through http://dda.dk/catalogue/22248 (accessed on 31 May 2021). Archive number: 22248.

## Supplementary Materials

The following are available online at https://www.mdpi.com/article/10.3390/ijerph18116106/s1. Table S1: Fruit and/or vegetable intake (g/day) in groups of tertiles at baseline.
