# Supplementary material for: Longitudinal Associations between Intake of Fruit and Vegetables and Height Attainment from Preschool to School Entry
Source: Int J Environ Res Public Health. 2021 Jun 5;18(11):6106. doi: 10.3390/ijerph18116106 (PMC8201233; doi:10.3390/ijerph18116106)
Supplement: Supplementary file 1 [file ijerph-18-06106-s001.zip › ijerph-1159368-supplementary.pdf]

Supplementary

Table S1. Fruit and/or vegetable intake (g/day) in groups of tertiles at baseline

	All n=553	Boys N=314	Girls N=239
Fruit intake (g/day), mean (SD)			
low	37.6 (17.0)	38.4 (17.4)	36.8 (16.7)
moderate	85.6 (13.5)	85.8 (13.4)	85.3 (13.8)
high	160.8 (45.7)	162.9 (47.6)	157.8 (42.8)
Vegetables intake (g/day), mean (SD)			
low	40.1 (17.9)	37.6 (18.3)	43.9 (16.7)
moderate	88.6 (13.4)	88.3 (13.5)	89.1 (13.3)
high	164.4 (52.4)	160.4 (49.8)	169.5 (55.6)
F&V intake (g/day), mean (SD)			
low	101.8 (33.1)	103.9 (34.0)	98.9 (31.7)
moderate	179.3 (20.9)	180.3 (20.1)	178.2 (22.0)
high	295.9 (63.7)	296.5 (63.9)	295.1 (63.9)
